# Multi-level cellular and functional annotation of single-cell transcriptomes using scPipeline

**DOI:** 10.1038/s42003-022-04093-2

**Published:** 2022-10-28

**Authors:** Nicholas Mikolajewicz, Rafael Gacesa, Magali Aguilera-Uribe, Kevin R. Brown, Jason Moffat, Hong Han

**Affiliations:** 1 Donnelly Centre, University of Toronto, Toronto, ON Canada; 2 Program in Genetics and Genome Biology, The Hospital for Sick Children, Toronto, ON Canada; 3 Department of Molecular Genetics, University of Toronto, Toronto, ON Canada; 4 Institute for Biomedical Engineering, University of Toronto, Toronto, ON Canada

**Keywords:** Computational platforms and environments, Gene regulatory networks, Statistical methods, Functional clustering, Gene expression

## Abstract

Single-cell RNA-sequencing (scRNA-seq) offers functional insight into complex biology, allowing for the interrogation of cellular populations and gene expression programs at single-cell resolution. Here, we introduce scPipeline, a single-cell data analysis toolbox that builds on existing methods and offers modular workflows for multi-level cellular annotation and user-friendly analysis reports. Advances to scRNA-seq annotation include: (i) co-dependency index (CDI)-based differential expression, (ii) cluster resolution optimization using a marker-specificity criterion, (iii) marker-based cell-type annotation with Miko scoring, and (iv) gene program discovery using scale-free shared nearest neighbor network (SSN) analysis. Both unsupervised and supervised procedures were validated using a diverse collection of scRNA-seq datasets and illustrative examples of cellular transcriptomic annotation of developmental and immunological scRNA-seq atlases are provided herein. Overall, scPipeline offers a flexible computational framework for in-depth scRNA-seq analysis.

## Abstract (web summary)

scPipeline is a single-cell data analysis toolbox that builds on existing methods and offers modular workflows for multi-level cellular annotation and user-friendly analysis reports.

## Introduction

Single-cell RNA-sequencing (scRNA-seq) has facilitated the characterization of multi-cellularity at unprecedented resolution, with the advancement of high-throughput protocols enabling profiling experiments that include millions of cells in a single experiment. While experimental protocols such as SMART-seq2^1^, Drop-seq^2^, sci-RNA-seq3^3^ and commercial 10X genomics vary in approach and scale, gene expression matrices (gene-by-cell count) are ultimately generated and represent a common starting point for most downstream analyses.

The development of computational toolboxes like Seurat^4–7^, Scanpy^8^, and Cell Ranger (10X Genomics, commercial) facilitates scRNA-seq analyses broadly across a diverse array of research topics. These tools offer application-tailored functionalities, including data pre-processing, normalization, quality control (QC) and clustering analysis. However, comprehensive analyses still require a degree of computational expertise. With the more recent emergence of interactive and notebook-based analysis platforms, scRNA-seq analysis has become more accessible to users lacking high-level computational skills^9–11^. However, despite the user-friendly interface offered by these platforms, difficulties can arise with custom-tailored analyses, or when data integration between different scRNA-seq platforms is required, a practice that is becoming more routine as complimentary and comparable datasets emerge. To address these limitations, we have developed scPipeline, a report-based single-cell analytic toolbox. scPipeline is offered as a series of Rmarkdown scripts that are organized into analysis modules that generate curated reports. The modular framework is highly flexible and does not require complete reliance on a single analysis platform. Additionally, the self-contained reports generated by each module provide a comprehensive analysis summary and log of analytic parameters and scripts, thereby ensuring reproducible and shareable analysis workflows.

In tandem to scPipeline, we developed the scMiko R package that comprises a collection of functions for application-specific scRNA-seq analysis and generation of scPipeline analytic reports. We validate proposed scRNA-seq methods implemented in scMiko that facilitate multi-level cellular and functional annotation. Specifically, using eight reference scRNA-seq datasets, we validate the co-dependency index (CDI) as a differential expression (DE) method that identifies binary differentially-expressed genes (bDEGs), propose a specificity-based resolution criterion to identify optimal cluster configurations, describe the Miko scoring pipeline for cell-type annotation, and introduce scale-free shared nearest neighbor network (SSN) analysis as a gene program discovery method.

The scMiko R package (https://github.com/NMikolajewicz/scMiko) and scPipeline scripts (https://github.com/NMikolajewicz/scPipeline) are available on GitHub. Step-by-step tutorials and documentation are also provided at https://nmikolajewicz.github.io/scMiko/.

## Results

### Overview of scPipeline modules

**Fig. 1:**
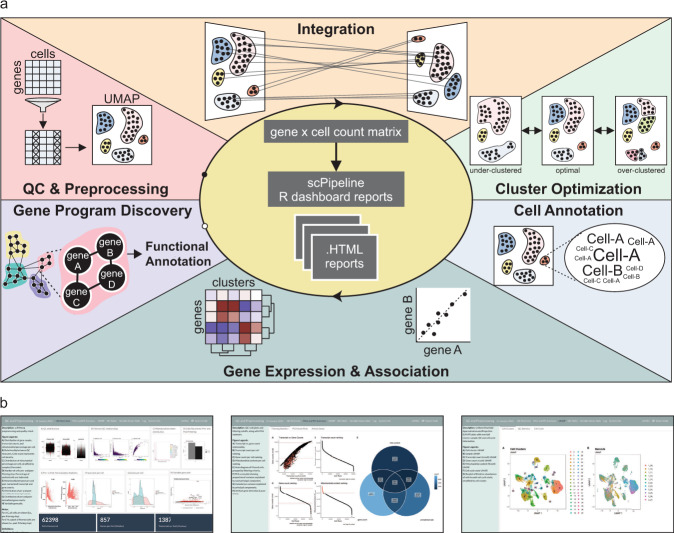
Schematic of scPipeline analysis modules. **a** scPipeline is a modular collection of Rmarkdown scripts that generate reports for scRNA-seq analyses. The modular framework permits flexible usage and facilitates i) QC & preprocessing, ii) integration, iii) cluster optimization, iv) cell annotation, v) gene expression and association analyses, and vi) gene program discovery. Each standalone HTML report provides a comprehensive analysis summary that can be seamlessly shared without any dependencies. Alternatively, online repositories (e.g., GitHub) can be used to host HTML reports for public dissemination. **b** Representative snapshots of scPipeline reports generated using the QC and preprocessing (*left*), cell annotation (*middle*), and gene expression and association (*right*) modules. More examples can be found here.

Here we introduce scPipeline, a modular collection of R markdown scripts that generate curated analytic HTML reports for scRNA-seq analyses (Fig. 1a). For a given gene expression matrix, the *QC and preprocessing module* performs data filtering (based on mitochondrial content and gene recovery) and normalizes the count matrix using the scTransform algorithm implemented in Seurat^12^. The module outputs a Seurat object (for downstream analyses), and a corresponding standalone HTML report that summarizes the results^13^ (Fig. 1b). In the case of multiple scRNA-seq datasets (e.g., experimental replicates, multiple studies and/or public datasets), we provide an *integration module* that leverages the canonical correlation analysis and reciprocal principal component analysis approaches implemented in Seurat to facilitate data integration for downstream analyses^5^. Once data has been preprocessed, cells are clustered using the *cluster optimization module*, where we introduce a specificity-based criterion for identifying the optimal resolution for Louvain community-based clustering. For each candidate cluster resolution, we also report DEGs identified using the Wilcox and CDI DE methods, for which we highlight specific and distinct applications in our current work. Once the optimal cluster configuration has been identified, the annotation modules facilitate cell type and cell state annotation using a priori cell-type markers, analysis of gene expression and associations, and unsupervised gene program discovery and functional annotation. Notably, the *cell annotation module* utilizes our gene set scoring method (i.e., the Miko score) to reliably annotate cell clusters using cell-type-specific markers. The Miko score is distinct from existing gene set scoring methods in that it adjusts for inherent variations in gene set size, thereby enabling direct comparison and ranking of gene set scores computed across gene sets of varying size. To facilitate gene expression exploration, we also developed a *gene expression and association module* which enables users to explore the expression pattern of query genes and predict gene function based on gene co-similarity profiles. Similarity profiles can be constructed using various methods, including Spearman correlation, rho proportionality, and CDI metrics^14^. These profiles are then functionally annotated to identify putative pathways correlated with the gene of interest. Finally, the *gene program discovery module* is used for gene program detection and transcriptomic network visualization. In addition to providing validated gene program discovery methods (e.g., ICA and NMF), we introduce the SSN method, which we demonstrate has superior recovery of known gene ontologies (GO) and enrichment of STRING-curated protein-protein interactions (PPI). Collectively, scPipeline offers a streamlined and reproducible workflow with user-friendly and intuitive reports and contributes to the current computational resources available for scRNA-seq. Importantly, its modular framework provides a foundation upon which future analysis modules can be developed to support additional scRNA-seq analyses.

### Co-dependency index identifies cell-type specific markers

**Fig. 2:**
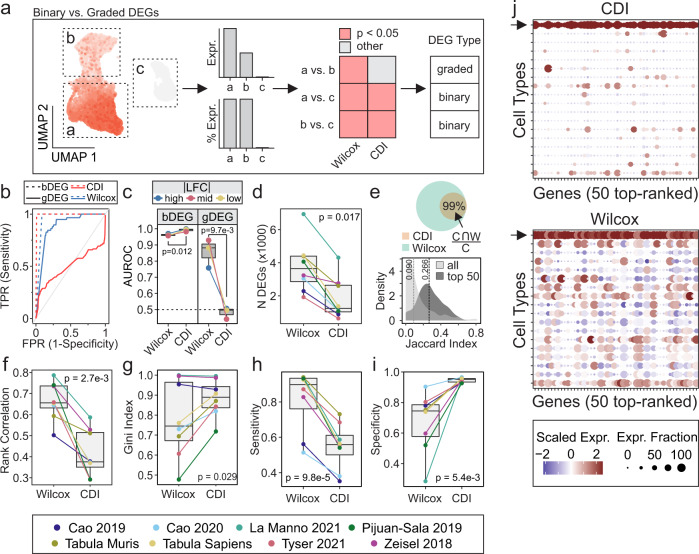
Co-dependency index identifies cell-type specific markers. **a** Schematic illustrating binary and graded DEGs in scRNA-seq analysis. For a given cellular population, the UMAP shows gene expression, barplots summarize expression (Expr.) and percentage of expressing cells (% Expr.) across subpopulations, and heatmap compares how Wilcoxon and CDI methods can be applied to classify genes as graded or binary DEGs. **b**, **c** Performance of Wilcoxon and CDI methods in recovering bDEGs and gDEGs from simulated scRNA-seq data was evaluated using ROC analysis and representative ROC curves (**b**) and comparisons between methods (**c**) are shown. Simulated scRNA-seq data were simulated with varying LFC magnitudes (*see methods*). For **b**, *red dashed curve*: CDI bDEG recovery, *red solid curve*: CDI gDEG recovery, *blue dashed curve*: Wilcoxon bDEG recover, *blue solid curve*: Wilcoxon gDEG recovery. For **c**, *blue*: high |LFC|, *red*: mid |LFC|, *yellow*: low |LFC|. **d**–**i** DEGs were identified by Wilcoxon and CDI methods across eight public scRNA-seq datasets and evaluated for number of significant DEGs (5% FDR, **d**), DEG overlap (**e**), rank correlation of average gene expression with -log10(*p*) values (**f**), Gini inequality index (**g**), sensitivity (**h**), and specificity (**i**). For **h**–**i**, sensitivity and specificity refer to the fraction of expressing cells within and outside of a cluster, respectively, thus serving as descriptive characterization of each method rather than how sensitive/specific each method is in detecting the true set of DEGs. For **e**, the *top panel* shows overlap between DEGs identified by Wilcoxon (W, *green*) and CDI (C, *orange*), whereas the *bottom panel* shows the distribution of Jaccard similarities across all significant DEGs (*light gray*) and top 50 DEGs (*dark gray*). For **g**–**i**, the top 50 DEGs identified by each method were considered. **j** Representative dot plot of top 50 DEGs identified by CDI (*top*) and Wilcoxon (*bottom*) methods in yolk sac mesoderm cell population from Tyser 2021 scRNA-seq data (*arrows* indicate row corresponding to the yolk sac mesoderm population). For all comparisons, *p* values were determined by paired Wilcoxon ranked sum test. Matched pairs are illustrated in each boxplot using connecting lines and represent instances where Wilcoxon and CDI were both applied to the same dataset. bDEG binary DEG, CDI co-dependency index, DEGs differentially-expressed genes, FDR false discovery rate, gDEG graded DEG, ROC receiver operating characteristic.

Robust identification of DEGs between cell populations is critical in scRNA-seq analyses. DEGs can be further subclassified into two different groups: graded DEGs (gDEG), in which genes are expressed in both populations, but to varying degrees; and bDEG, in which genes are exclusively expressed in one population but not the other (Fig. 2a). Popular scRNA-seq DE methods, such as the Wilcoxon method^15^, identify DEGs indiscriminately and require additional downstream filters to parse out bDEGs. Thus, a method tailored towards specifying bDEGs is needed.

Here we propose using the CDI to identify cluster-specific bDEGs within scRNA-seq data. Using simulated and real scRNA-seq datasets (Table 1), we identified DEGs using the CDI and Wilcoxon methods, and then evaluated each method’s relative performance and behavior. With simulated data, we demonstrated that CDI selectively recovered bDEGs (AUROC = 0.982–0.999), but not gDEGs (AUROC = 0.398–0.507), in a manner that was independent of UMI:gene ratio, cell counts, and log-fold change (LFC) magnitudes (Fig. 2b, c, Supplementary Fig. 1a). In contrast, the Wilcoxon method recovered bDEGs (AUROC = 0.957–0.990) and gDEGs (AUROC = 0.619–0.929), and gDEG recovery was dependent on the magnitude of LFC between two groups (Fig. 2c, Supplementary Fig. 1a). Comparing the two methods, bDEG recovery was reliably recovered by both methods (AUROC > 0.95); however, CDI performed significantly better (Fig. 2c, *p* = 0.012) and Wilcoxon could not discriminate between bDEGs and gDEGs. We next extended our characterization of the CDI method to real scRNA-seq data. Here we observed that CDI method identified 66% fewer DEGs than the Wilcoxon method (1241 vs. 3653 genes, *p* = 0.017) (Fig. 2d). Among the CDI-derived DEGs, 99% were also recovered by the Wilcoxon method (Fig. 2e) indicating that the CDI method selectively identifies a subset of Wilcoxon-derived DEGs. Among all the significant DEGs obtained by either method, the median Jaccard similarity was 0.09; however, when only the top 50 DEGs [ranked by −log10(*p*)] were considered, the Jaccard similarity increased to 0.266, suggesting a bias towards bDEGs among top DEGs identified by Wilcoxon (Fig. 2e). We were also interested in determining whether at higher cluster resolutions the CDI DEG profile can discern between subpopulations. To address this, we performed DE analyses on data clustered at varying resolutions (Supplementary Fig. 1b, c). Despite higher cluster resolutions (i.e., more clusters) being associated with fewer DEGs per cluster, most datasets consistently had at least one significant CDI and Wilcoxon DEG (5% FDR) up to a resolution of 10. This signified that although the CDI method recovers fewer DEGs than Wilcoxon, the CDI DEG profile is still sufficient to characterize clusters at higher resolutions. Consistent with prior reports, the Wilcoxon method was systematically biased towards calling highly-expressed genes differentially-expressed (Fig. 2f)^15^. We next evaluated the cluster-discriminating characteristics of the top 50 genes identified by each method (Fig. 2g–i, Supplementary Fig. 1d, e; *see Methods* for definition of cluster-discriminating metrics). While the Wilcoxon method identified genes with higher cluster-discriminating sensitivity (0.90 vs. 0.56, *p* = 9.8e-5; Fig. 2h) and negative predictive value (NPV; 0.87 vs. 0.70, *p* = 1.2e-3; Supplementary Fig. 1d), the CDI method had superior specificity (0.95 vs. 0.75, *p* = 5.4e-3; Fig. 2g, i) and positive predictive value (PPV; 0.91 vs. 0.75, *p* = 7.6e-4, Supplementary Fig. 1e). As an illustrative example, we evaluated the top 50 DEGs in yolk-sac mesoderm, where we observed a higher degree of cluster-specificity among the top markers identified by the CDI method (Fig. 2j). Together our analyses establish that the CDI method selectively identifies bDEGs, compared to the Wilcoxon method that indiscriminately identifies bDEGs and gDEGs.

**Table 1:** Public scRNA-seq datasets used in the current study.

Dataset	Description	Species	Method	*N*		Analyses
				Cells (% subset)	Cell Types	
Tabula Muris^69^	Pan-atlas	Mm	10X	100,000 (99%)	100	A, B, C
Tabula Sapiens^60^	Pan-atlas	Hs	10X	100,000 (21%)	158	A, B, C
Cao 2019^3^	Organogenesis	Mm	sci-RNAseq3	50,000 (100%)	37	A, B, C, E
Cao 2020^70^	Fetus	Hs	sci-RNAseq3	100,000 (26%)	77	A, B, C, D, E
Pijuan-Sala 2019^21^	Gastrulation	Mm	10X	100,000 (77%)	38	A, B, C, D, E
Tyser 2021^17^	Gastrulation	Hs	SMART-seq2	1,195 (100%)	18	A, B, C, D, E
La Manno 2021^27^	Developing brain	Mm	10X	100,000 (39%)	16, 136	A, B, C, E
Zeisel 2018^61^	Adolescent brain	Mm	10X	22,238 (100%)	39	A, B, C, E
Han 2022^71^	neural differentiation	Mm	sci-RNAseq-3	26,117 (100%)	-	E
Ochocka 2021^25^	immune cells	Mm	10X	40,401 (100%)	-	E

Analyses in which the datasets were used are indicted as *A*: DE methods, *B*: cluster resolutions, *C*: cell type gene sets, *D*: Miko scoring, *E*: gene program discovery.

*Hs* Homo sapiens (human), *Mm* Mus musculus (mouse).

### Marker specificity-based criterion for identifying optimal cluster resolutions

**Fig. 3:**
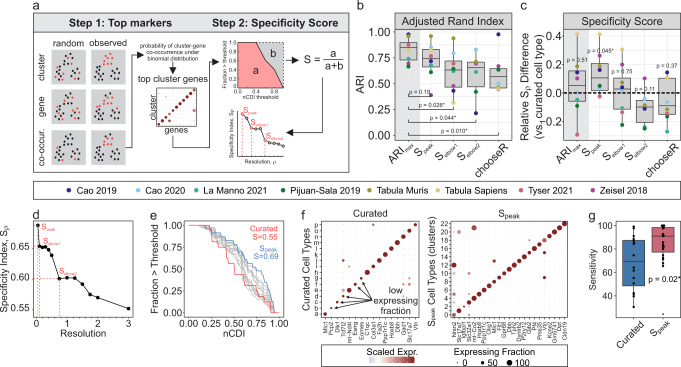
Identification of optimal clustering resolution using a specificity-based criterion. **a** Schematic of specificity-based resolution-selection criteria. **b**, **c** Adjusted Rand index (**b**) and specificity index differences (**c**) between ground truth (author-curated) clusters and observed Louvain clusters, using resolutions determined by different optimization criteria [specificity criteria (${S}_{{peak}},{S}_{{elbow}1},{S}_{{elbow}2}$) and *chooseR*^58^]. ${AR}{I}_{{\max }}$ represents resolution at which maximal ARI was achieved, after considering all candidate resolutions (0.5–3). For **b**, significance compared to *ARI*_max_determined by paired Wilcoxon test. For **c**, significance compared to zero (i.e., ground truth) was determined by one-sample Wilcoxon test. *N* = 8 independent scRNA-seq datasets. **d**–**g** Optimal clustering resolution for Tyser 2021 human gastrulation scRNA-seq data^17^. **d** Relationship between resolution and specificity indices, and identification of ${S}_{{peak}}$, ${S}_{{elbow}1}$ and ${S}_{{elbow}2}$. **e** Specificity-curves. *Gray curves*: all candidate resolutions evaluated (0.5–3), *blue curve*: S_peak_, *red curve*: ground truth (curated) clusters. **f** Dot plots of top markers for curated (*left*) and ${S}_{{peak}}$ (*right*) clusters. **g** Comparison of cluster sensitivity (expressing fraction) of each top marker obtained for curated and ${S}_{{peak}}$ clusters. Significance determined by unpaired Wilcoxon test.

scRNA-seq-based cell type identification relies on unsupervised clustering methods; however, resulting cell clusters can vary drastically depending on what resolution is used to perform clustering. Many approaches have been proposed to guide the selection of the optimal resolution, including silhouette index and resampling-based methods (e.g., chooseR and MultiK^16^). However, these methods are motivated by theoretical rather than biological criterion. Having demonstrated that the CDI method yields cluster-specific markers (Fig. 2), we propose to define cell types at a clustering resolution that maximizes the specificity of markers obtained in each cluster. We proceed by first clustering over a range of candidate resolutions, and the top specific marker in each cluster at each resolution is identified using the CDI method (Fig. 3a, *step 1*). Subsequently, specificity curves are generated for each resolution and used to obtain aggregate specificity metrics. The resolution at which maximal specificity is observed is taken as the optimal resolution, ${S}_{{peak}}$ (Fig. 3a, *step 2*).

Recognizing that there exist multiple resolutions that may be biologically relevant (e.g., cell types vs. cell subtypes), an approach to specifying the optimal set of resolutions that recovers this hierarchical system of cellular classification is warranted. As the cluster resolution increases and cell clusters are split into progressively smaller and more homogeneous subpopulations, differences between cell types will become smaller, as reflected by the incremental drops in the specificity metric along the specificity curve as resolution increases (Fig. 3a, *step 2*). We found that following such a drop, the specificity index proceeds to remain relatively stable over a range of resolutions, resulting in a characteristic “elbow” in the Specificity curve. We found that these elbows typically coincide with higher-resolution cluster configurations that reflect more resolved cell types, as showcased in the Pijuan-Sala murine gastrulation atlas (Supplementary Fig. 2), and thus we hypothesized that these “elbows” represent biologically relevant and stable clustering configurations, and termed these ${S}_{{elbow}1}$ and ${S}_{{elbow}2}$.

To evaluate the performance of our specificity-based resolution selection criteria (${S}_{{peak}}$, ${S}_{{elbow}1}$, and ${S}_{{elbow}2}$), we used eight public scRNA-seq datasets, and adopted author-curated cell types as “ground-truth” clusters. We showed that our specificity-based criteria favor clustering configurations that align with manually curated cluster labels, as indicated by the lack of significant difference between the adjusted Rand index (ARI; i.e., a measure of classification consistency) obtained at ${S}_{{peak}}$ and ${AR}{I}_{{\max }}$ resolutions (Fig. 3b). By comparison, *chooseR* (a resampling-based resolution selection criteria), ${S}_{{elbow}1}$ and ${S}_{{elbow}2}$ yielded clusters with significantly lower ARI, suggesting that these cluster configurations represent cell subtypes, whereas clusters obtained at the ${S}_{{peak}}$ resolution represent well-defined cell type clusters (Fig. 3b). In support of this, ${S}_{{peak}}$ clusters were associated with significantly more specific markers (i.e., top markers were more specific) than “ground truth” clusters (*p* = 0.045), whereas there was no significant difference observed for the other cluster configurations compared to “ground truth” clusters. As a representative example, we applied our specificity-based resolution selection approach to the human gastrulation scRNA-seq data published by Tyser et al.^17^ (Fig. 3d). Compared to curated clusters, ${S}_{{peak}}$ clusters were associated with a higher specificity index (0.69 vs. 0.56) (Fig. 3e) which was verified by visual inspection (Fig. 3f), and further, it was demonstrated that the top markers associated with ${S}_{{peak}}$ clusters were significantly more sensitive (i.e., high expression fraction; *p* = 0.02) than those obtained in “ground truth” clusters (Fig. 3g). Our results demonstrate that a specificity-based resolution selection criterion reliably identifies cluster configurations that reflect biologically relevant cell types.

### Marker-based cluster annotation with Miko score

**Fig. 4:**
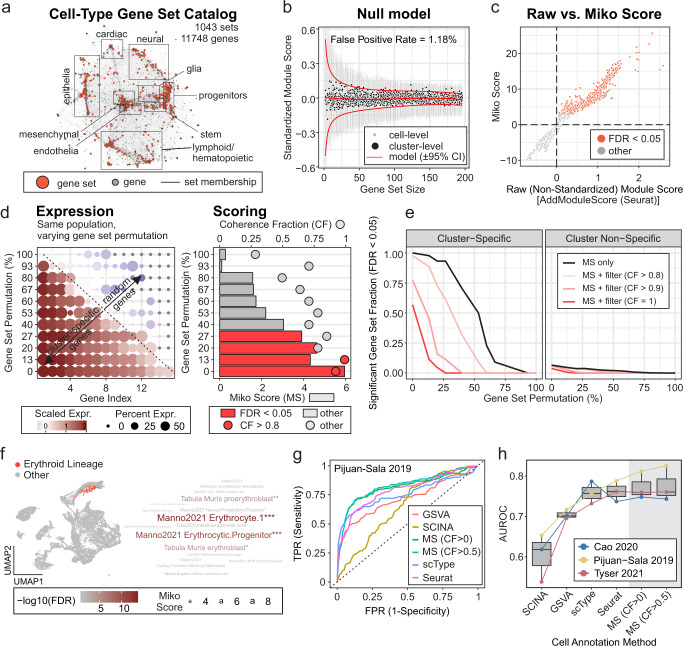
Cell-type annotation. **a** Cell-type-specific gene set catalog represented as bipartite network. Edges between gene sets (*red nodes*) and genes (*gray nodes*) represent gene set membership. Major cell ontologies are annotated, and the corresponding gene sets can be accessed using the scMiko R package. **b** Representative null model relating gene set size and standardized module scores (SMS; for random gene sets). *Red curves*: predicted mean SMS ± 95% CI; *black points*: observed cluster-level mean SMS; *gray points*: observed cell-level SMS. **c** Relationship between cluster-level non-standardized module score (AddModuleScore, Seurat R package) and Miko score. Clusters with significant module activity (FDR < 0.05) are indicated. **d**, **e** Evaluation of Miko score performance. **d** Representative gene sets with varying rates of permutation (i.e., substitution of cluster-specific gene with random gene; *left*) and corresponding Miko scores (bar plot, *right*) with coherent fractions (dot plot, *right*). **e** Relationship between degree of gene set permutation and fraction of cluster- and cluster-non-specific gene sets with significant (FDR < 0.05) module activity. Coherent fraction (CF) filters were included to demonstrate capacity to titrate score sensitivities and specificities. **f** Representative example of Miko score applied to murine gastrulation data^21^ using cell-type gene set catalog (**a**). UMAP illustrates cell population with curated cell-type of interest (erythroid lineage), and word cloud represents top cell types predicted by the Miko scoring algorithm. **g** Receiver operating characteristic (ROC) analysis illustrating cell annotation performance of marker-based scoring algorithms applied to murine gastrulation atlas^21^. **h** Cell annotation performance of Miko Scoring compared to existing marker-based scoring algorithms. Boxplots illustrate area under receiver operating characteristic (AUROC) curves across different methods and data sets. Methods were rank ordered by average AUROC performance.

Transcriptome-wide expression profiling has led to the generation and availability of gene sets for cell-type identification. Nonetheless, the external validity of these genes sets is remarkably inconsistent, largely stemming from the fact that many gene sets are derived using one-versus-all DE methods on genetic backgrounds that lack population-level phenotypic diversity. While elucidating the exact conditions under which a gene set reliably identifies a given cell type is beyond the scope of the current study, we argue that cell-type specific gene sets obtained using one-versus-all DE methods are most valid when derived from diverse cell atlases. To complement our marker-based cluster annotation efforts, we performed DE analysis on the eight public scRNA-seq datasets presented in Table 1, each comprising highly diverse cell types. Together with cell type markers reported in Zhao 2019^18^ and the PanglaoDB, we provide a catalog of cell type markers comprising 1043 (redundant) cell type-specific marker sets spanning 11748 unique genes (Supplementary Data 1, Table S1). Representing the cell-type marker catalog as a bipartite network revealed major cell type hubs including epithelial, mesenchymal, endothelial, and lymphoid/hematopoietic cell types, in addition to tissue-specific cell ontologies like cardiac, neural, and glial cells (Fig. 4a).

Many marker-based cell annotation methods have been described;^19, 20^ however, one limitation of these methods is a lack of consideration for gene set size. As the number of genes in a gene set increases, pooled signature scores become less sensitive to the influence of highly expressed individual genes. This gene set size dependency leads to a bias, such that scores obtained from smaller gene sets tend to have more spurious enrichments than those obtained from larger gene sets (Fig. 4b), precluding unbiased comparison of signature scores obtained over a range of unevenly sized gene sets. Motivated by this limitation, we introduce the Miko score, a cell cluster scoring method that accounts for variations in gene set sizes. The Miko score also provides a hypothesis-testing framework capable of rejecting non-significantly enriched gene sets (Fig. 4). For a given single-cell dataset, query and size-matched random gene sets are scored using a standardized implementation of AddModuleScore(…), and the difference between query and random module scores is scaled using the size-adjusted standard deviation estimate obtained from a gene set size-dependent null model (Fig. 4b) to yield the Miko score (Fig. 4c). The standardized implementation of AddModuleScore(…) accounts for cell-to-cell variation in gene expression, while scaling by the size-adjusted standard deviation estimate adjusts for size-related dependencies and results in a test statistic from which a *p* value can be derived.

The performance of Miko score-based cell annotation was evaluated using cell-type-specific gene sets derived for each cell type in the mouse gastrulation dataset reported by Pijuan-Sala et al.^21^. To assess the robustness of the Miko score and account for inaccuracies in gene set definitions, each set was permuted to varying extents, such that a subset of cell-type specific markers in each gene set were replaced with an equal number of randomly sampled genes (Fig. 4d). Using non-permuted gene sets, the Miko score-based enrichments were 100% sensitive and 94% specific for cluster-specific gene sets (Fig. 4e). When 25% of genes were permuted, we observed 93% sensitivity and 96% specificity. However, at higher permutation rates, we observed a significant decline in sensitivity such that at 50% permutation there was 54% sensitivity and 98% specificity. We also found that filtering enrichments using a coherence criterion resulted in marginally improved specificity at the cost of sensitivity (Fig. 4e). As an illustrative example, we calculated Miko scores using our cell-type marker catalogue (Fig. 4a; Pijuan-Sala-derived markers were omitted to minimize overfitting) and demonstrated that author-curated erythroid (Fig. 4f) and endoderm (Supplementary Fig. 3a) populations were accurately annotated using our Miko score pipeline. To benchmark the Miko scoring method against existing marker-based scoring algorithms, including SCINA^22^, GSVA^23^, scType^24^, and Seurat, a selection of scRNA-seq datasets were scored and predicted cell annotations were compared to the author-curated labels (i.e., ground truth) through ROC analysis (Fig. 4g, Supplementary Fig. 3b, c). We found that the Miko scoring method, coupled with post-hoc coherence filtering (AUROC = 0.776), yielded superior results to GSVA (AUROC = 0.705) and SCINA (AUROC = 0.603), and marginally better annotations than the Seurat (AUROC = 0.762) and scType (AUROC = 0.759) algorithms (Fig. 4h). Collectively, our analyses establish the Miko score as a marker-based scoring algorithm that is robust to gene set inaccuracies and capable of facilitating unbiased comparison across a large collection of unevenly sized gene sets.

### Gene program discovery using scale-free topology shared nearest network analysis

**Fig. 5:**
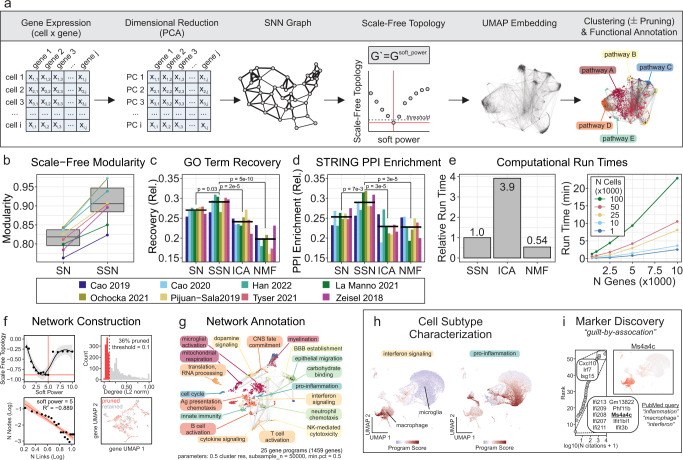
Gene program discovery using scale-free shared nearest neighbor network (SSN) analysis. **a** Schematic illustrating network construction and annotation. **b** Network modularity with (SSN) and without (SN) scale-free topology enforcement. **c**–**e** Comparison of GO term recovery (**c**), STRING PPI enrichment (**d**) and computational run time (**e**) across different gene program discovery methods. ICA; independent component analysis, NMF; non-negative matrix factorization, SN; shared nearest neighbor network, SSN; scale-free shared nearest neighbor network. (**f**–**i**) Representative transcriptional network construction, annotation and applications using Ochocka 2021 scRNA-seq data^25^. **f** Optimal soft power required for scale-free topology (*left column*; threshold = −0.9) and pruning of genes with low network connectivity (*right column*; threshold = 0.1). **g** Functional annotation of gene programs. GO term enrichment was performed using hypergeometric overrepresentation analysis. **h** Activity of “interferon signaling” and “pro-inflammation” programs overlaid on cell UMAP. Macrophage and microglial subpopulations can be subtyped by program activity status. **i** Marker discovery and functional prediction using guilt-by-association. Genes belonging to “interferon signaling” program were cross-referenced with PubMed articles queried using “inflammation”, “macrophage” and “interferon” search strings to identify candidate genes (e.g., Ms4a4c) implicated in interferon signaling. Ms4a4c expression was visualized on a UMAP to verify that expression is coherent with gene program activity.

Unsupervised gene program discovery offers a complementary approach to annotating cell clusters in scRNA-seq, which aim to group genes based on co-expression similarity profiles. Here we introduce the SSN method to identify gene expression programs (Fig. 5a). In brief, the gene expression matrix is dimensionally reduced using principal component analysis (PCA). Each gene’s K-nearest neighbors (KNN) are then determined by Euclidean distance in PCA space. The resulting KNN graph is used to derive a shared nearest neighbor (SNN) graph by calculating the neighborhood overlap between each gene using the Jaccard similarity index. Adopting the framework from weighted gene correlation network analysis (WGCNA), an adjacency matrix that conforms to a scale-free topology is then constructed by raising the SNN graph to an optimized soft-thresholding power, which effectively accentuates the modularity of the network (Fig. 5b). The resulting adjacency matrix is used to construct the network UMAP embedding and to cluster genes into programs (or modules) by Louvain community detection [the optimal clustering configuration is determined using a purity-based criterion (Supplementary Fig. 4)]. To reduce noise, genes with low connectivity (i.e., low network degree) are pruned so that only hub-like genes are retained for downstream annotation and analysis.

Compared to independent component analysis (ICA) and non-negative matrix factorization (NMF), SSN gene programs had significantly superior GO term recovery and STRING PPI enrichment (Fig. 5c, d). The importance of enforcing a scale-free topology was evident in the comparison between SN (shared nearest neighbor network *without* scale-free topology) and SSN (shared nearest neighbor network *with* scale-free topology) (Fig. 5c, d). On average, the relative computational runtimes were 0.54, 1, and 3.9 for NMF, SSN, and ICA methods, respectively, thereby establishing NMF as the fastest algorithm, but only by a small margin over SSN which significantly outperformed ICA (Fig. 5e). Nonetheless, the SSN method can infer the gene association network for 50,000 cells and 5000 genes in just under 5 min (Fig. 5e)

We demonstrated the use of SSN gene program discovery and network visualization with two case examples (Fig. 5f–l). In the first case, we constructed an SSN network using scRNA-seq data of the murine immune compartment in brains engrafted with the syngeneic GL261 GBM cell line^25^ (Fig. 5f). Functional annotation of each gene program revealed a diverse transcriptomic landscape (Fig. 5g), including interferon signaling and pro-inflammatory programs that were highly active in monocyte/macrophage and microglial sub-populations, respectively (Fig. 5h). In addition to facilitating further cellular characterization, functionally annotated gene programs offer opportunities to predict the function of previously uncharacterized genes using a “guilt-by-association” approach. For example, cross-referencing genes belonging to the interferon-signaling gene program in the SSN graph with PubMed-indexed publications, we find the gene *Ms4a4c* had not been previously associated with “inflammation”, “macrophage” or “interferon”. The membrane-spanning 4A (MS4A) family is conserved in vertebrates and includes 18 members with a tetraspan structure in humans^26^. MS4A family members are differentially and selectively expressed in immunocompetent cells such as B cells (CD20/MS4A1) and macrophages (MS4A4A), associate and modulate the signaling activity of different immunoreceptors, and have been linked to different pathological settings including cancer, infectious disease and neurodegeneration^26^. We predict that *Ms4a4c*, a previously uncharacterized mouse gene, may have a role in the macrophage-related inflammatory process (Fig. 5i).

In our second case example, we demonstrate how SSN gene program discovery can identify and facilitate the refinement of robust gene signatures (Supplementary Fig. 5). Briefly, we constructed a SSN network from scRNA-seq data derived from a murine developing brain^27^ (Supplementary Fig. 5a, b) and show that the expression of each gene belonging to the angiogenesis program is positively correlated with the aggregate gene program score when examined in the developing murine brain data from which the signature was derived (Supplementary Fig. 5c). Notably, in two other independent datasets (murine and human gastrulation), only a subset (albeit majority) of genes were positively correlated with the program score (Supplementary Fig. 5c). By taking the 3-way intersection of coherent genes across these three relevant datasets, we find a 64-gene signature (Supplementary Fig. 5d) that was specifically enriched among the hematogenic endothelial populations in all three scRNA-seq datasets (Supplementary Fig. 5e).

## Discussion

We have described a pair of computational resources, scMiko (R package) and scPipeline (dashboard analysis reports), and propose new methods to facilitate multiple levels of cluster annotation in scRNA-seq data. Our computational tools follow established scRNA-seq analytic practices, and offer modular workflows that enable data preprocessing, normalization, integration, clustering, annotation, gene program discovery and gene association analyses. Among the methods proposed in this work, we validated the CDI as a DE method that identifies binary DEGs. Given the inherent specificity of bDEGs, we then adopted the CDI algorithm to derive a specificity-based resolution selection criterion for determining optimal clustering configurations and benchmarked the performance of this approach against ground truth annotations. Upon identifying the optimal cluster resolution(s), we demonstrate how to annotate clusters using our Miko Scoring pipeline, which facilitates unbiased scoring of a diverse set of variable-sized cell type-specific gene sets and accepts or rejects candidate annotations using a hypothesis-testing framework. Finally, we describe SSN analysis as an approach to identify and functionally annotate gene sets in an unsupervised manner, providing an additional layer of functional characterization of scRNA-seq data.

There are several existing interactive single-cell analytic frameworks available including Cellar^28^, SingleCAnalyzer^29^, and ICARUS^30^, and each offers its own unique advantages including user-friendly interfaces and cloud computing, which make single-cell analyses accessible to the research community. While scPipeline offers many of the same functionality (e.g., data preprocessing, integration and annotation), it is distinguished from prior frameworks through its portability, customizability, and modular design. Each scPipeline module is designed to generate a self-contained HTML report that serves as a portable record of analysis that can be shared and disseminated without any external dependencies. Furthermore, since each module is provided as a Rmarkdown script, more advanced R users have the option to customize analyses to their specifications, whereas online tools are often limited to a prespecified set of functions that cannot be modified by the user. Finally, the modular design of scPipeline means that users can plug in their data anywhere along the analytic pipeline, thereby allowing for seamless integration across different analytic workflows. Thus, we envision scPipeline as a toolbox that complements existing frameworks and offers the research community with additional flexibility to analyze single-cell data.

The annotation methods presented here, namely finding bDEGs with CDI, cell-type annotation with Miko Scoring, and gene program discovery and functional annotation with SSN analysis, all complement and expand the extensive list of analysis methods for scRNA-seq^31, 32^. It has become evident from systematic benchmarking efforts that no single method is enough to probe single-cell datasets in-depth, and that different methods offer unique advantages with regards to biological accuracy, interpretability, computational complexity, visualization, or accessibility^14, 15, 33^.

Reliable annotation begins with identifying the optimal clustering configuration. Although there are many ways to cluster single-cell data including K-means (SAIC, RaceID3), hierarchical (CIDR, BackSPIN^34^, SINCERA) and density-based (Monocle2, GiniClust^35^) clustering approaches, we used the community-detection based Louvain approach implemented in Seurat due to its low run time and high performance index^36, 37^, and focused on optimizing the resolution that controls the number of resolved clusters. If cells are clustered at an inappropriately low resolution (i.e., under-clustered), there is a risk of amalgamating distinct cell types into single populations, resulting in a loss of resolution in cellular identity. In contrast, if the resolution is too high (i.e., over-clustered), multiple near-identical cellular lineages emerge and obscure the true complexity of the dataset. Nevertheless, it is recognized that clustering configurations at multiple different resolutions may be biologically meaningful, and reflect different layers of cellular identities, such as cell types at lower resolutions (e.g., macrophage), and cellular sub-types (M1 vs. M2 polarized macrophage) at higher resolutions^16^. There are different selection criteria for identifying the optimal resolution(s), including the silhouette index and resampling-based methods (e.g., chooseR, MultiK^16^); however, these methods are motivated by theoretical rather than biological criterion. The specificity-based resolution selection criterion described in our current work identifies cluster configurations coinciding with maximal marker specificity. This is a desirable property for downstream applications that require individual biomarkers to resolve cell types, such as flow cytometry or imaging. Additionally, when evaluated over multiple candidate resolutions, more than one biologically relevant resolution is often identified, manifesting as “elbows” on the specificity-resolution curve (i.e., akin to the elbow method used for selecting the number of principal components on a Scree plot). We benchmarked the performance of our specificity-based criterion against author-curated “ground truth” annotations and demonstrated that a specificity-based criterion outperforms the resampling-based approach used in chooseR. We note that a limitation of our method relates to the stability and reproducibility of clusters, especially in single-replicate data sets. Artifact genes (i.e., genes that are highly expressed exclusively in a small subset of cells belonging to a single experimental replicate) have been shown to produce distinct cellular clusters; in the absence of experimental replicates it is difficult to determine whether these clusters represent technical artifacts or real biology^38, 38^. While this can be addressed by sampling multiple biological replicates^38^, it may also be circumvented by expanding our specificity-based criterion to consider the top 5–10 markers, rather than the top single cluster-specific marker at each resolution. Finally, although we evaluated our specificity-based criterion using the Louvain clustering approach, the criterion may be applied to any clustering method that requires optimization of the number of resolved clusters (e.g., K-means). We expect that our specificity-based criterion will complement existing optimization methods to find meaningful cluster configurations.

The CDI DE method offers an approach to identifying bDEGs, which have applications distinct from gDEGs. Whereas gDEGs are useful for identifying differences that occur on a spectrum (e.g., neural development), bDEGs have greater utility in identifying cell-type-specific markers (e.g., FACS sorting of CD34^+^ for hematopoietic stem cells), diagnostic biomarkers, disease targets (e.g., CART-cell therapy), and artifact genes in scRNA-seq datasets^38^. A known limitation of existing DE methods for scRNA-seq is the failure to account for variation in biological replicates, and the CDI approach is no exception^15^. Nonetheless, we expect that with appropriate biological replicates and external validation, the CDI DE method will contribute to the identification of specific biomarkers.

The Miko scoring cell-type annotation workflow described in this work supplements the existing repertoire of marker-based annotation algorithms including scCatch^39^, SCSA^40^, SCINA^22^, and CellAssign^41^. The hypothesis testing framework implemented in the Miko scoring pipeline enables the rejection of unlikely cell-type annotations, a property that is shared by SCINA and CellAssign. However, unlike its predecessors, Miko scoring explicitly corrects for gene set size biases, thereby enabling unbiased comparison of scores over a large collection of various-sized gene sets. This property enables prioritization of the most likely annotation if multiple marker sets are significantly scored for a given population. Coupled with our word cloud-based visualizations introduced in scMiko and scPipeline, candidate cell-type annotations can be easily examined.

To facilitate marker-based annotation of cell types, several reference databases are available including CellMatch, CellMarker, PanglaoDB, CancerSEA, and MSigDB (collection 8)^42^. We contribute to these resources by deriving marker sets from diverse single-cell atlases (Table 1), and through network-based visualization we demonstrate the hierarchical complexity of cell ontology (Fig. 4a). While the network organization was generally coherent with the cell-type annotations assigned to the marker sets, an inspection of select local neighborhoods in our cell-type marker network revealed occasional co-similarities between marker sets from heterogeneous cell types, reflecting either inaccuracies in marker curation or similarities in cellular processes across dissimilar cell types. Based on these observations, we emphasize that marker-based annotations are only as good as the cell-type prescribed to the original dataset. Thus, integrating a large collection of marker sets from multiple independent sources to achieve consensus annotations, or alternatively, using a robustly validated collection of marker sets can attain optimal results.

The SSN method for gene program discovery was inspired by the established SNN framework used in single-cell analyses to reliably identify cell-to-cell distances in a sparse dataset, as well as the scale-free topology transformation used under the assumption that the frequency distribution of gene association in a transcriptomic network follows the power law^43–45^. A UMAP-embedded network, based on a SNN graph akin to that used in our SSN procedure, has previously resolved gene modules corresponding to protein complexes and pathways, with Euclidean distances in UMAP space out-performing correlation and PCA distances in predicting protein-protein interactions^46^. Consistent with these findings, we demonstrated that gene programs identified by SSN yielded superior GO term recovery and enrichment of STRING PPIs compared to ICA and NMF methods, and that the scale-free topology transform was critical in driving this improvement in performance. Taken together, the SSN gene program discovery method is robust to data sparsity, has a high-performance index, offers network-based visualization, and has run-times that scale well for larger datasets.

Future plans for scPipeline and scMiko involve continual review and improvement of existing workflows, as well as development and/or implementation of new methods that facilitate complementary analyses such as characterization of ligand-receptor interactions^47, 48^, regulon-based transcription factor inference^49^, trajectory analyses^3, 50, 51^ and differential-abundance analyses^52^. As innovative approaches to interrogate single cell data are proposed by us and others, we will continue to build this “open” resource.

## Methods

### Software

Figure preparation: CorelDRAW x8 (Corel); Bioinformatic analyses: R v 4.0.3 (R Foundation for Statistical Computing).

### Computational resources

Analyses were run on a desktop computer with an Intel Core i9-10900L CPU (3.70 GHz, 10 cores, 20 threads) with 120 GB RAM running Windows 10 Pro (v21H2).

### Data preprocessing

scRNA-seq data sets were normalized, scaled, dimensionally reduced and visualized on a UMAP using the *Seurat* (v 4.0.4) workflow^4–7^. In brief, count matrices were loaded into a Seurat object and normalized using *NormalizeData*(…, normalization.method = “LogNormalize”, scale.factor = 10,000). Variable features were identified using *FindVariableFeatures* (…, selection.method = ‘mvp’, mean.cutoff = c(0.1,8), dispersion.cutoff = c(1,Inf)) and then data were scaled using *ScaleData*(…). Principal component analysis, and UMAP embedding was performed using *RunPCA*(…) and *RunUMAP*(…, dims = 1:30), respectively. Metadata from original publications were used to annotate cell types.

### Differential expression analysis

Differential expression analyses were performed using Wilcoxon rank sum (Wilcox) and codependency index (CDI)^53, 54^. The Wilcox method was implemented using the *wilcoxauc* function (*Presto* R package, v 1.0.0)^55^. Alternatively, the CDI was adopted to calculate the probability of cluster and gene co-occurrence under a binomial distribution. For a given gene *g* and cluster *k*, the joint probability of observed non-zero *g* expression in *k* is formulated as:

1 $$P(\,g=1,k=1)=P(\,g=1)P(k=1)={\pi }_{g,k}$$

The probability of observing a test statistic more extreme under the null hypothesis that gene *g* and cluster *k* are independent is then:

2 $${p}_{e}({\pi }_{g,k})=\mathop{\sum}\limits _{{{\mathbb{I}}}_{{{{{{\rm{g}}}}}},{{{{{\rm{k}}}}}}}\le {{{{{\rm{x}}}}}}\le {{{{{\rm{N}}}}}}}{{{{{\boldsymbol{Bino}}}}}}(N,x,{\pi }_{g,k})$$

where ${{{{{\boldsymbol{Bino}}}}}}(N,x,{\pi }_{g,k})$ represents the probability of observed *x* successes in *N* trials if the probability of success is ${\pi }_{g,k}$, and ${{\mathbb{I}}}_{{{{{{\rm{g}}}}}},{{{{{\rm{k}}}}}}}$ is the number of cells in which *g* and *k* are coincident. CDI is then defined as:

3 $${CDI}=-{{{\log }}}_{10}[{p}_{e}({\pi }_{g,k})]$$

We further normalized the CDI score using the CDI score corresponding to the probability of observed a perfect co-dependency for cluster *k*:

4 $${nCDI}=\frac{{CDI}}{-{{{\log }}}_{10}[{p}_{e}({\pi }_{k,k})]}$$

where ${\pi }_{k,k}=P\left({c}_{k}=1,{c}_{k}=1\right)$, under the assumption of independence. Possible values of ${nCDI}$ range between [0,1], such that ${nCDI}=1$ represents perfect co-dependence between a gene and cluster, and ${nCDI}=0$ represents no co-dependence but is not equivalent to mutual exclusivity which has been formulated elsewhere^54^. The CDI DE method is implemented in R using the *findCDIMarkers*(…) function in the scMiko package.

Since the CDI metric is influenced by the degree of sparsity in the single cell count matrix, scoring data sets with varying sequencing depths (i.e., different degrees of drop out) will yield heterogeneous CDI results. Thus, if using an integrated Seurat object, heterogeneity in sequencing depth can be corrected by processing the Seurat object using Seurat’s *PrepSCTFindMarkers*(…) function which effectively down samples the count matrix to a homogenous sequencing depth across all datasets. As a complementary approach, we have also provided the *findConservedCDIMarkers*() function in the scMiko package [analogous to Seurat’s *FindConservedMarkers*() function] that finds gene markers that are conserved across independent groups by pooling the p values from independent group-specific estimates using Fisher’s method^56, 57^.

The CDI, by definition, only computes genes that are “up-regulated” relative to the comparison group, so to ensure fair comparison to the Wilcox method, only gene subsets that had a positive log fold change (LFC) were considered in Wilcox vs. CDI comparative analyses. Differentially expressed genes (DEGs) were deemed significant at a 5% false discovery rate (FDR). The top 50 DEGs identified by each method were subsequently characterized using sensitivity, specificity, positive predictive value (PPV) and negative predictive value (NPV):

5 $${Sensitivity}=\frac{{P}_{{in}}}{\left(100 \% -{P}_{{in}}\right)+{P}_{{in}}}$$

6 $${Specificity}=\frac{100 \% -{P}_{{out}}}{\left(100 \% -{P}_{{out}}\right)+{P}_{{out}}}$$

7 $${PPV}=\frac{{P}_{{in}}}{{P}_{{in}}+{P}_{{out}}}$$

8 $${NPV}=\frac{100 \% -{P}_{{out}}}{\left(100 \% -{P}_{{out}}\right)+(100 \% -{P}_{{in}})}$$

where ${P}_{{in}}$ and ${P}_{{out}}$ represent the expressing percentage of cells within and outside a cluster, respectively. Note that these metrics are intended as a descriptive characterization of each DE method, rather than to measure of how sensitive/specific each method is in detecting the true set of differential genes. We also computed the Gini inequality index as a complementary surrogate for gene specificity:

9 $${Gini}\,{Index}=\frac{{\sum }_{k}^{{n}_{{clusters}}}\left(1-\left({x}_{g,k}-{{\max }}\left({x}_{g}\right)\right)\right)}{{n}_{{clusters}}-1}$$

where ${x}_{g,k}$ is the average expression of gene *g* for cluster *k*, and ${n}_{{clusters}}$ is the number of unique clusters.

To benchmark and compare the performance of CDI and Wilcoxon methods, scRNA-seq datasets were simulated using *splatSimulate*(…) (*Splatter* R package, v1.20.0) with nine sets of parameters (Table S2), each with two cluster groups. The simulation parameters were varied to reflect differences in sequencing depth (UMI:gene ratio), number of cells, and magnitude of difference (i.e., LFC magnitude). True DEGs were defined as any feature with a Splatter DEFacGroup2 value not equal to 1. Half of the true DE genes were then assigned as bDEGs by setting the count values of genes outside of Group2 to zero, and the remaining half were specified as gDEGs. For each dataset, Wilcoxon and CDI methods were run using *getDEG*(group.by = “Group”, …) and *findCDIMarkers*(features.x = “Group”, features.y = rownames(seurat_object), …) functions, respectively, from the scMiko R package using default parameters. Performance was evaluated using ROC analysis where CDI and Wilcoxon recovered DEGs were compared to true DEGs simulated by Splatter. ROC sensitivity and specificity were calculated using confusionMatrix(…) (*caret* R package, v6.0-92).

### Cluster optimization

To identify the optimal cluster resolution, we first clustered samples over a range of candidate resolutions (0.05 to 3) using *FindClusters*(…, algorithm = 1) in Seurat. At each resolution *ρ*, the top cluster-specific marker for each cluster was identified using CDI-based DE analysis. Subsequently, specificity curves were generated by plotting the proportion of clusters that exceed a threshold nCDI score, for nCDI ranging [0,1]. The area under this curve (AUC) represents the aggregate specificity index ${S}_{\rho }$ and possible values range between [0,1], with a score of 1 representing the ideal cluster configuration in which each cluster has at least one marker satisfying nCDI = 1. Aggregate specificity indices were graphed over the range of candidate resolutions, and resolutions at which a peak and subsequent elbow(s) were manually observed were taken as optimal clustering resolutions for downstream analyses. Cluster resolutions were also identified using *chooseR* algorithm with default parameters (https://github.com/rbpatt2019/chooseR)^58^.

For each resolution, we computed the adjusted Rand index (ARI) between unsupervised scRNA-seq clusters and author-curated cell-type clusters (i.e. ground truth) using the *adj.rand.index* (*fossil* R package, v 0.4.0)^59^. ARI is a measure of similarity between two data clusterings, adjusted for chance groupings. Across all the candidate resolutions evaluated, the maximal ARI between our unsupervised clusters and ground truth clusters was ~0.8 and the resolutions at which the max ARI was observed was denoted ${AR}{I}_{{\max }}$ (Fig. 3b). The imperfect cluster similarity here reflects differences in computational preprocessing across datasets and possible manual cluster refinement performed by authors of the original datasets. Nonetheless, this represents the maximal ARI that is achievable using the current unsupervised cluster approach and serves as a positive control to which all other cluster configurations were compared.

### Cell-type marker catalog

To generate a cell-type marker reference catalog, cell-type-specific markers were derived from eight diverse public scRNA-seq atlases (Tabula Muris, Tabula Sapiens^60^, Cao 2019^3^, Cao 2020, Pijuan Sala^21^, Tyser, La Manno^27^, and Zeisel^61^) using the Wilcoxon DE method to identify DEGs across author-curated cell types (Table 1). All markers satisfying logFC > 0.5, AUROC > 0.95 and FDR < 1% were included. If less than 15 markers were identified per a cell-type using these criteria, the top N markers (ranked by logFC) with FDR < 1% were taken to ensure the minimum 15 markers per cell-type requirement was satisfied. These markers were then consolidated with cell-type-specific markers from PanglaoDB and CellMarkers^18^ to yield a cell-type marker reference catalog. No additional filtering was performed, resulting in many cell-types being represented by multiple gene sets from several independent sources. We justified this redundancy as a strength of the catalog, as co-enrichment of independent and coherent cell-type terms leads to higher confidence cell-type annotations. To visualize the catalog using a bipartite network, a gene *×* cell-type incidence matrix was generated using *graph.incidence* (*igraph* R package, v 1.2.6) and the network was visualized using *layout.auto* (*igraph*). Both human and murine cell-types are represented in this catalog. All cell-type markers used in this study have been made available in our scMiko R package and Table S1.

### Cell-type annotation

The Miko score is a scaled cluster-level module score that adjusts for cell-to-cell gene expression variation and gene set size. To compute the Miko score, standardized module scores ${Z}_{j}$ for each cell *j* must first be calculated by subtracting the mean expression of control features ${Y}_{j}$ from the mean expression of gene set features ${X}_{j}$, and then scaling the difference by the pooled standard deviation of the gene set and control features:

10 $${Z}_{j}=\frac{{X}_{j}-{Y}_{j}}{\sqrt{{Var}({X}_{j})+{Var}({Y}_{j})}}$$

Following the approach taken by Tirosh and colleagues^62^ and implemented in *AddModuleScore* (Seurat), all analyzed features are binned based on averaged expression and control features are randomly selected from each bin. As a variance-corrected statistic, the standardized module score can be used as-is to compute single-cell level significance [$p={{\Pr }}\left( > \left|Z\right|\right)$]. However, in the absence of a gene set-size correction, module score comparisons between gene sets are invalid.

To correct for gene set size-dependencies, cell-level null standardized module scores ${Z}_{{null},j}$ are computed for randomly sampled gene sets that span over a range of different sizes (2-100 genes per gene set by default). Random gene set-specific ${Z}_{{null},j}$ scores are then aggregated for each cluster *k* to yield a cluster-level null standardized module score ${Z}_{{null},k}$:

11 $${Z}_{{null},k}=\frac{1}{{n}_{{cell},k}}\left(\mathop{\sum }\limits_{j}^{{n}_{{cell},k}}{Z}_{{null},j}\right)$$

where ${Z}_{{null},k}$ and ${Z}_{{null},j}$ represent the null standardized module scores for a randomized gene set of a given size for cluster *k* or cell *j*, respectively, and ${n}_{{cell},k}$ represents the number of cells belonging to cluster *k*. The relationship between gene set size and null standardized scores is then fit using a polynomial spline:

12 $${null}{{{{{\rm{\_}}}}}}{mode}{l}_{{mean}}={{{{{\rm{glm}}}}}}\left({Z}_{{null}} \sim {bs}\left({size},{degree}=3,{family}={gaussian}\right)\right)$$

This null mean model is used to predict gene set size-adjusted null standardized scores ${Z}_{{null}}^{{pred}}$. In theory, the expected value of ${Z}_{{null}}^{{pred}}$ is 0 and we approximate it as such in our computational implementation. Separately, we calculate the observed variance in ${Z}_{{null},k}$, denoted ${Var}({Z}_{{null},k})$, over a range of gene set sizes, and fit the relationship between gene set size and ${Var}({Z}_{{null},k})$ using a gamma-family generalized linear model:

13 $${null}{{{{{\rm{\_}}}}}}{mode}{l}_{{variance}}={{{{{\rm{glm}}}}}}\left({Var}\left({Z}_{{rand}}\right) \sim {size},{family}={Gamma}\right)$$

This null variance model is used to predict gene set size-adjusted variance of standardized scores ${Var}({Z}_{{null}}^{{pred}})$.

Finally, to derive the gene set-size corrected Miko score, we aggregate standardized module scores ${Z}_{j}$ for each gene set into cluster-level means:

14 $${Z}_{{obs},k}=\frac{1}{{n}_{{cell},k}}\left(\mathop{\sum }\limits_{j}^{{n}_{{cell},k}}{Z}_{j}\right)$$

and center and scale ${Z}_{{obs},k}$ using gene set-size matched null mean ${Z}_{{null}}^{{pred}}$ and variance ${Var}({Z}_{{null}}^{{pred}})$ to yield the Miko score ${M}_{k}$ for cluster *k*:

15 $${M}_{k}=\frac{{Z}_{{obs},k}-{Z}_{{null}}^{{pred}}}{\sqrt{{Var}({Z}_{{null}}^{{pred}})}}$$

The Miko score is a cluster-level module score that is adjusted for gene set size-related spurious effects and cell-to-cell variability. This ensures the valid comparison of scores across differently sized gene sets, making it a valuable tool in marker-based cell annotation. Another property of the Miko score is that it can be handled as a Z statistic, thus facilitating p-value calculation and hypothesis testing:

16 $$p={{\Pr }}\left( > \left|{M}_{k}\right|\right)$$

This facilitates cell cluster annotation based on which cell-type-specific gene sets are significantly active.

In addition to the Miko score, we propose two post-scoring filters which serve to fine tune which gene sets are considered enriched. The first is a coherence filter in which a positive correlation between component gene expression and the Miko score is enforced for a minimum fraction of component genes. The second is a frequent flier filter, which flags gene sets that exceed a minimum significance rate and represent gene sets that enrich across most cell clusters.

To benchmark the performance of Miko scoring, three public scRNAseq datasets (Cao 2020, Tyser 2021 and Pijuan Sala 2019; *see* Table 1) were annotated using Miko scoring, SCINA^22^, scType^24^, Seurat (*AddModuleScore* function), and GSVA^23^. Each dataset was annotated using our cell marker catalog (Table S1) excluding the marker sets that were derived from the dataset being annotated to prevent overfitting. Miko scoring was performed with default parameters, with [MS (CF>0.5)] and without [MS (CF>0)] the coherence filter (CF). SCINA was performed using *SCINA*(rm_overlap = 0, …) using otherwise default parameters (*SCINA* R package, v 1.2.0). GSVA was performed with default parameters using *gsva*(…) (*GSVA* R package, v 1.44.2). Seurat scoring was performed using *AddModuleScore*(…) with default parameters (*Seurat* R package, v 4.0.4). ScType scoring was performed using *sctype_score*(…) using default parameters and the script provided on github (https://github.com/IanevskiAleksandr/sc-type/). Gene set-specific scores from scType, Seurat, GSVA, and SCINA were then averaged over each cell cluster and performance was evaluated using ROC analysis using author-curated labels as ground-truths. ROC sensitivity and specificity were calculated using *confusionMatrix*(…) (*caret* R package, v 6.0-92).

### Gene program discovery

Scale-free topology *s*hared nearest neighbor *n*etwork (SSN) analysis is a gene program discovery algorithm that groups genes based on co-expression similarity profiles and visualizes the network layout using a UMAP-based embedding. Features used for gene program discovery can be pre-specified using a variety of criteria, including minimum expression thresholds, high variability or deviance, however in the current study we select features using a minimal expression criteria (expressing fraction >0.5 within at least one cluster). The cell × gene expression matrix (transposed from the Seurat object) is then subject to principal component analysis [*RunPCA*(…, ndim = 50)] and the top components explaining >90% of the variance are used to construct a K-nearest neighbor graph *K* [*FindNeighbors*(…, k.param = 20)], from which a shared-nearest neighbor (SSN) graph *G* is constructed by calculating the neighborhood overlap (Jaccard Index) between every gene and its K-nearest neighbors. Adopting the framework from WGCNA, a scale-free topology transform is then applied to the SNN graph by raising the SNN graph (gene × gene matrix) to an optimized soft-threshold power:

17 $$G^{\prime} ={G}^{{soft}{{{{{\rm{\_}}}}}}{power}}$$

where $G^{\prime}$ represents a scale-free topology-conforming SNN graph and is the adjacency matrix that will be used for downstream network construction. The optimal soft-threshold power used to derive $G{\prime}$ is identified by calculating the signed ${R}^{2}$ statistic for the following relationship:

18 $${{\log }}\left(p\left(W\right)\right) \sim {{\log }}\left(W\right)$$

where *W* represents connectivity *w* discretized into *n* bins (default 20), and $p(W)$ represents the proportion of nodes (i.e., genes) within the *W* bin. Connectivity ${w}_{g}$ for gene *g* is calculated as row-wise sum of *G*:

19 $${w}_{g}=\sum {G}_{g,-g}$$

where *g* and $-g$ represent the row and column indices corresponding to gene *g* and all genes except gene *g*, respectively. The soft threshold power is evaluated over a range of candidate values (default 1–5), and the optimal power is taken as the smallest power for which signed ${R}^{2} < -0.9$:

20 $$\mathop{{{\arg }}\,{{\min }}}\limits_{{{soft}\; {power}}\in [0.5,5]}\left({{{{{\rm{signed}}}}}}\,{R}_{{soft} \; {power}}^{2} < -0.9\right)$$

To visualize the transcriptomic network, the scale-free SNN graph $G{\prime}$ is embedded in a UMAP using *RunUMAP*(…, graph = $G$′, umap.method = “umap-learn”). Network nodes represent individual genes, whereas network linkages represent $G^{\prime}$ edges thresholded at a specified quantile (0.9 by default).

To identify gene programs from the scale-free SNN graph $G^{\prime}$, Louvain clustering is performed. We identify the optimal clustering resolution using *a nearest neighbor purity criterion* which seeks to optimize the cluster consistency, or purity, within individual gene neighborhoods by maximizing the similarity of genes within programs compared to other programs (analogous to silhouette score^63^). For a candidate cluster resolution *ρ*, the gene-level purity score is defined as the proportion of genes within gene *g*’s neighborhood that belong to the most represented cluster within that neighborhood (Supplementary Fig. 5):

21 $${p}_{\rho ,g}=\frac{|{k}_{\rho ,g}\in {{{{{\rm{mode}}}}}}({k}_{\rho ,g})|}{|{K}_{g}|}=\frac{{n}_{\rho ,g}}{{N}_{g}}$$

where ${p}_{\rho ,g}$ is the gene $g$’s purity at *ρ* resolution, the denominator ${N}_{g}$ represents the cardinality $({||})$, or size, of gene $g$’s K-nearest neighborhood ${K}_{g}$ (20 by default), the numerator ${n}_{\rho ,g}$ represents the number of genes in gene $g$’s neighborhood that belong to the most represented cluster [i.e., majority cluster, ${{{{{\rm{mode}}}}}}({k}_{\rho ,g})$] and ${k}_{\rho ,g}$ is a vector of cluster memberships for all genes belonging to gene $g$’s neighborhood. For each candidate resolution, gene-level purity scores ${p}_{\rho ,g}$ are then aggregated as means to yield the global purity score ${P}_{\rho }$:

22 $${P}_{\rho }=\frac{1}{N}\mathop{\sum}\limits _{g}{p}_{\rho ,g}$$

where *N* is the number of genes in the SSN graph. Finally, the optimal cluster resolution is the maximal resolution at which the target purity ${P}_{{target}}$ (0.8 by default) is satisfied:

23 $$\mathop{{{\arg }}\,{{\max }}}\limits_{\rho \,\in [0,{{{{{\rm{\infty }}}}}}]}(|{P}_{{target}}-{P}_{\rho }|)$$

Possible purity scores range between 0 to 1. Neighborhoods in which genes belong to many different clusters are considered “impure” (low purity score) whereas neighborhoods in which genes belong to a single cluster are “pure” (high purity score). In general, higher cluster resolutions are associated with lower the purity scores, however we recommend using a target purity between 0.7 (more gene programs) and 0.9 (fewer programs).

To minimize spurious gene program associations, genes with low connectivity (i.e., low network degree) are pruned so that only hub-like genes are retained for downstream annotation and analysis. Here connectivity for each gene *g* is calculated as described above for ${w}_{g}$, however in this case we use the scale-free SSN graph $G^{\prime}$ instead of *G*. Connectivity scores ${w}_{i}$ are L2 normalized and those below a prespecified threshold (0.1 by default) are pruned.

### SSN performance evaluation

To benchmark the performance of SSN, gene program discovery was performed using SSN, ICA, and non-negative matrix factorization (NMF) on eight public scRNA-seq data sets (Table 1). The ICA method was selected for comparison because it was the top performing algorithm in a prior systematic benchmarking review of 42 routinely used gene program discovery algorithms^33^, whereas NMF is a popular discovery algorithm used in scRNA-seq analyses^64, 65^. For each dataset, a common subset of genes that was expressed by >50% of cells in at least one cell cluster were used (typically ranging between 1000 and 4000 genes). ICA was performed using *RunICA*(…) implemented in *Seurat* (default parameters), and NMF was performed using *nnmf*(…, *k* = c(5, 10, 15), loss = “mse”, rel.tol = 1e-4, max.iter = 50) (*NNLM* R package, v 0.4.4). For NMF analysis, scaled gene expression values were truncated at zero. *Graph modularity* was compared between SSN graphs before (SN) and after (SSN) scale-free topology transformation using *modularity*(…) (*igraph* R package, v 1.2.6). *GO* g*ene set recovery* was evaluated following the approach taken by Saelens and colleagues, where the Jaccard similarity between observed (SSN, ICA, NMF) and known (GO) gene programs was calculated to yield an observed *×* known gene program similarity matrix. Then, for each known gene program (matrix column), the max column-wise Jaccard similarity score was taken, representing the best recovery achieved by the unsupervised gene program detection algorithm for that known gene program, and the best Jaccard indices averaged over all known programs yielded the overall recovery score. The overall recovery score was compared across gene program detection methods. To evaluate the extent of *STRING protein-protein interaction enrichment* in gene programs identified by each method, within-program interaction enrichment was performed using *get_ppi_enrichment*(…) (*STRINGdb* R package, v 2.0.2) and enrichment ratios were compared across gene program discovery methods^66^. Finally, we used the murine gastrulation scRNA-seq data set to benchmark the *computing times* required to run each method. The data set was subsampled to 1000, 10000, 25000, 50000 and 100000 cells and for each data subset, 500, 1000, 2500, 5000, and 10000 genes were used for gene program discovery. The run times, relative to SSN, as well as the absolute run times for SSN across different cell/gene count settings were reported.

### Gene set enrichment analysis

To functionally annotate gene programs identified by SSN, ICA, and NMF, we perform hypergeometric overrepresentation analysis using *fora* (*fgsea* R package, v 1.14.0)^67^. Annotated gene sets used for enrichment analyses included GO ontology (biological processes, cellular components, molecular functions) and gene-set collections curated by the Bader Lab^68^.

### Data visualization

Unless otherwise specified, the *ggplot2* R package (v 3.3.5) was used for data visualization. scRNA-seq gene expression was visualized using *FeaturePlot* function (*Seurat*) or *DotPlot* function (*Seurat*). Venn diagrams were generated using either *ssvFeatureEuler* (*seqsetvis* R package, v 1.8.0) or *ggVennDiagram* (*ggVennDiagram* R package, v 1.1.4). Box plots are comprised of center line, median; box limits, upper and lower quartiles; whiskers; 1.5x interquartile ranges; points, raw data.

### Statistics and reproducibility

All pairwise comparisons were performed using the signed Wilcoxon rank sum test (two-sided), and *p* values were adjusted for multiple comparisons using the Benjamini–Hochberg procedure, as indicated. In cases where methods were compared across a common set of data, paired Wilcoxon tests (two-sided) were performed. Sample sizes reflected the number of scRNA-seq datasets evaluated, and not number of cells per dataset (see Table 1).

### Reporting summary

Further information on research design is available in the Nature Research Reporting Summary linked to this article.

## Supplementary information

Supplementary Information

Description of Additional Supplementary Files

Supplementary Data 1

Reporting Summary

## Data Availability

scRNA-seq data from Ochocka et al. (2021) was obtained from Gene Expression Omnibus (GEO; accession number GSE136001);^25^ Cao et al. (2019) from GEO (accession number GSE119945);^3^ Cao et al. 2020 from GEO (accession number GSE156793); Zeisel et al. (2018) from http://mousebrain.org/adolescent/downloads.html; La Manno et al. (2021) from http://mousebrain.org/development/downloads.html; Tabula Muris from FigShare; Tabula Sapiens from FigShare; Pijuan-Sala (2019) from the MouseGastrulationData R Package; and Tyser et al. (2021) from http://www.human-gastrula.net/^17^. Data and scripts used to generate figures have been deposited on figshare (10.6084/m9.figshare.21202757.v1).

## Code availability

scMiko R package (10.5281/zenodo.7111634) and scPipeline (10.5281/zenodo.7111632) are freely available and documentation and tutorial vignettes can be found here: https://nmikolajewicz.github.io/scMiko/.
