# Supplementary material for: Multi-level cellular and functional annotation of single-cell transcriptomes using scPipeline
Source: Commun Biol. 2022 Oct 28;5:1142. doi: 10.1038/s42003-022-04093-2 (PMC9616830; doi:10.1038/s42003-022-04093-2)
Supplement: Supplementary file 1 — Supplementary Information [file 42003_2022_4093_MOESM1_ESM.pdf]

Supplemental Figures

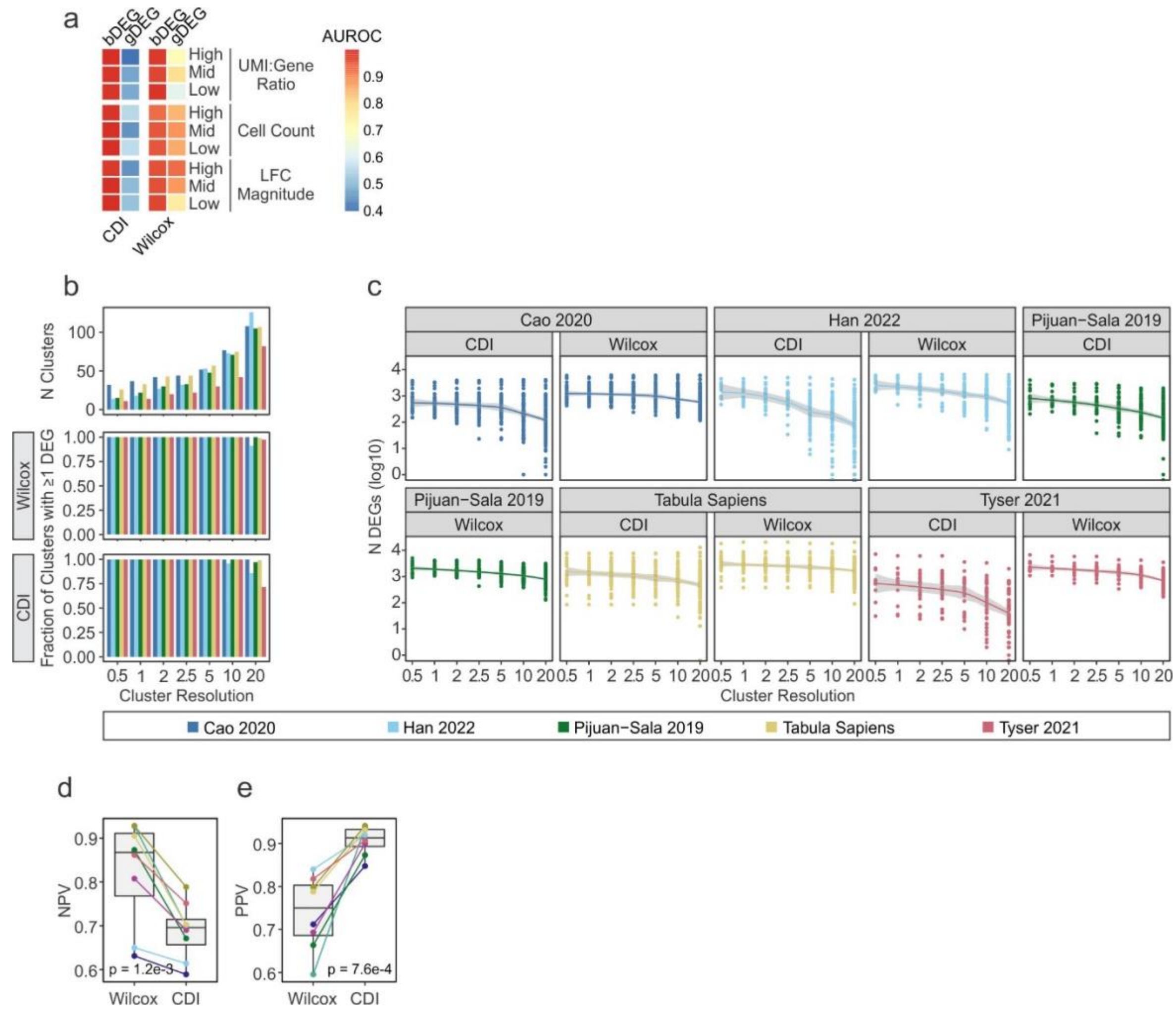

Supplemental Figure 1. DEG performance analysis. **(a)** Graded DEGs (gDEGs) and binary DEGs (bDEGs) were identified in simulated scRNA-seq sets using Wilcoxon and CDI methods and then compared against the ground truth using AUROC metric. Simulated datasets were generated using varying UMI:gene ratios, cell counts, or LFC magnitudes (*see methods*). **(b, c)** Wilcox and CDI analysis was performed on public scRNA-seq data that had been clustered at 0.5, 1, 2, 2.5, 5, 10, and 20 resolutions and the number of clusters and fraction of clusters with at least one DEG (5% FDR) were evaluated (**b**) and the association between cluster resolution and number (N) of DEGs across each dataset was determined (**c**). **(d, e)** DEGs were identified by Wilcoxon and CDI methods across eight public scRNA-seq datasets and evaluated for negative predictive value (NPV, **d**) and positive predictive value (PPV, **e**). AUROC; area under receiver operating characteristic curve, CDI; co-dependency index, DEGs; differentially-expressed genes.

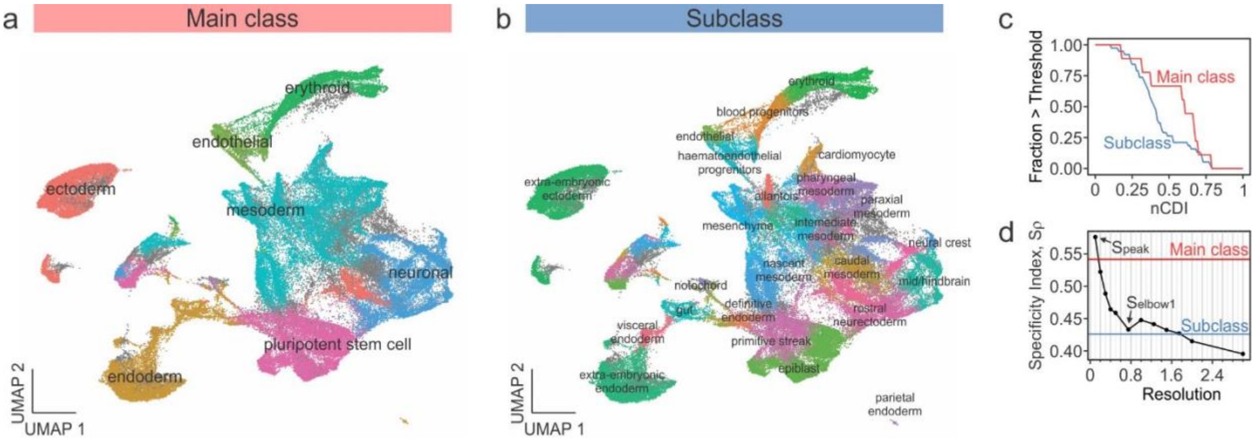

Supplemental Figure 2. Comparison of cell types annotations with optimized cluster configurations. **(a-b)** UMAPs of author-curated annotated murine gastrulation atlas (Pijuan Sala 2019) showing main classes (**a**) and subclasses (**b**). **(c)** Specificity-curve for main cells (*red curve*) and subclasses (*blue curve*) shown in **a**, **b**. **(d)** Relationship between cluster resolution and specificity indices. *Arrows*: S_{peak} and S_{elbow1}, *Horizontal lines*: Specificity indices corresponding to author-curated main classes (*red*) and subclasses (*blue*).

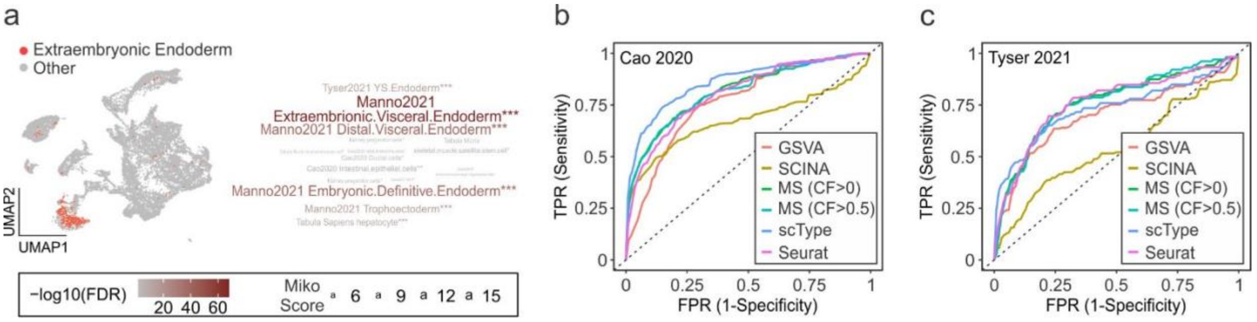

Supplemental Figure 3. Cell type annotation performance. **(a)** Representative example of Miko score applied to murine gastrulation data[24] using cell-type gene set catalog. UMAP illustrates cell population with curated cell-types of interest (Extraembryonic endoderm), and word clouds represent top cell types predicted by the Miko scoring algorithm. **(b-c)** Receiver operating characteristic (ROC) curves illustrating cell annotation performance of marker-based scoring algorithms applied to Cao 2020 (**b**) and Tyser 2021 (**c**) datasets (**Table 1**).

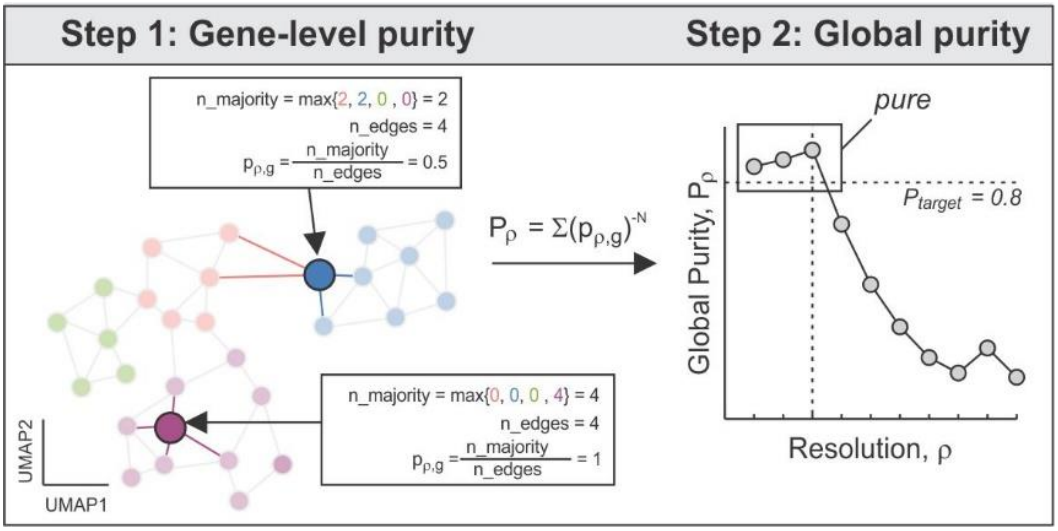

Supplemental Figure 4. Schematic of nearest neighbor purity criterion. The nearest neighbor purity criterion seeks to optimize the cluster consistency, or purity, within individual gene neighborhoods by maximizing the similarity of genes within gene programs compared to other programs. In step 1 (*left*), for a given cluster resolution ρ, the gene-level purity score $p_{\rho,g}$ is defined as the proportion of genes within gene g's neighborhood that belong to the most represented cluster within that neighborhood. The gene-level purity scores $p_{\rho,g}$ are then aggregated as means to yield the global purity P_{ρ}. In step 2 (*right*), the optimal cluster resolution (*vertical dashed line, right*) is the maximal resolution at which the target purity P_{target} is satisfied (0.8 by default; *horizontal dashed line, right*).

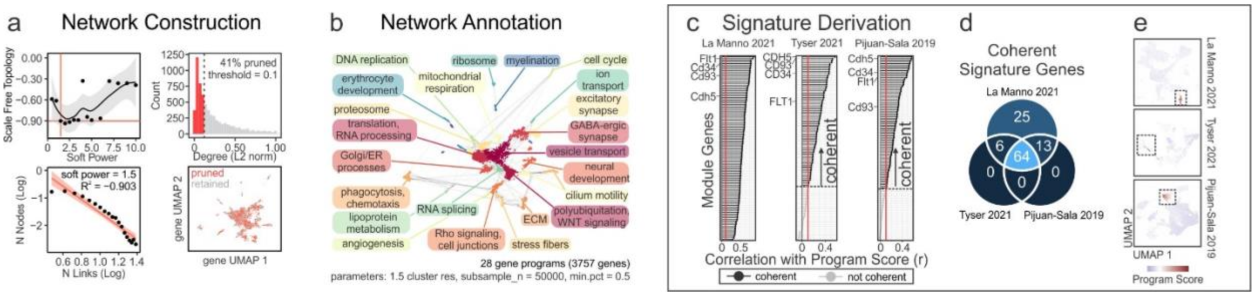

Supplemental Figure 5. Application of SSN to identify robust angiogenesis-associated gene program. (a-e) Representative transcriptional network construction, annotation and applications using La Manno 2021 scRNA-seq data[31]. **(a)** Optimal soft power required for scale-free topology (*left column*; threshold = 0.9) and pruning of genes with low network connectivity (*right column*; threshold = 0.1). **(b)** Functional annotation of gene programs. **(c-e)** External validation and refinement of angiogenesis signature. **(c)** Correlation between angiogenesis program activity and expression of individual gene program genes across three independent scRNA-seq datasets. Genes that exceeded the coherence threshold (Spearman correlation > 0.1) were deemed coherent. (**d**) Venn diagram illustrating intersection between coherent gene sets determined in each scRNA-seq dataset. 64/108 genes (59%) were coherent in all scRNA-seq datasets. **(e)** Gene program activity of coherent angiogenesis signature specifically highlights the (hematogenic) endothelial population in all three scRNA-seq datasets.

Supplemental Tables

Table S1. Catalog of cell type markers (Supplementary Data 1)

Table S2. Parameters used to simulate scRNA-seq datasets with Splatter[81].

Dataset	Parameters
Low UMI:Gene ratio	*newSplatParams*(nGenes = 5000, batchCells = 2000, group.prob = c(0.5, 0.5), dropout.type = "experiment", dropout.mid = -2, dropout.shape = 3, de.prob = 0.05, de.facScale = 0.4)
Medium UMI:Gene ratio	*newSplatParams*(nGenes = 5000, batchCells = 2000, group.prob = c(0.5, 0.5), dropout.type = "experiment", dropout.mid = 2, dropout.shape = 3, de.prob = 0.05, de.facScale = 0.4)
High UMI:Gene ratio	*newSplatParams*(nGenes = 5000, batchCells = 2000, group.prob = c(0.5, 0.5), dropout.type = "experiment", dropout.mid = 2, dropout.shape = 0.75, de.prob = 0.05, de.facScale = 0.4)
Low Cell Count	*newSplatParams*(nGenes = 5000, batchCells = 2000, group.prob = c(0.5, 0.5), dropout.type = "none", de.prob = 0.05, de.facScale = 0.4)
Medium Cell Count	*newSplatParams*(nGenes = 5000, batchCells = 5000, group.prob = c(0.5, 0.5), dropout.type = "none", de.prob = 0.05, de.facScale = 0.4)
High Cell Count	*newSplatParams*(nGenes = 5000, batchCells = 10000, group.prob = c(0.5, 0.5), dropout.type = "none", de.prob = 0.05, de.facScale = 0.4)
Low LFC Magnitude	*newSplatParams*(nGenes = 5000, batchCells = 2000, group.prob = c(0.5, 0.5), dropout.type = "none", de.prob = 0.05, de.facScale = 0.1)
Medium LFC Magnitude	*newSplatParams*(nGenes = 5000, batchCells = 2000, group.prob = c(0.5, 0.5), dropout.type = "none", de.prob = 0.05, de.facScale = 0.5)
High LFC Magnitude	*newSplatParams*(nGenes = 5000, batchCells = 2000, group.prob = c(0.5, 0.5), dropout.type = "none", de.prob = 0.05, de.facScale = 1.5)

LFC; log fold-change, UMI; unique molecular identifier
