# Supplementary material for: Multi-level cellular and functional annotation of single-cell transcriptomes using scPipeline
Source: Commun Biol. 2022 Oct 28;5:1142. doi: 10.1038/s42003-022-04093-2 (PMC9616830; doi:10.1038/s42003-022-04093-2)
Supplement: Supplementary file 2 — Description of Additional Supplementary Files [file 42003_2022_4093_MOESM2_ESM.pdf]

Description of Additional Supplementary Files

File name: Supplementary Data 1
Description: Catalog of cell type markers.
