# Supplementary material for: Multi-level cellular and functional annotation of single-cell transcriptomes using scPipeline
Source: Commun Biol. 2022 Oct 28;5:1142. doi: 10.1038/s42003-022-04093-2 (PMC9616830; doi:10.1038/s42003-022-04093-2)
Supplement: Supplementary file 4 — Reporting Summary [file 42003_2022_4093_MOESM4_ESM.pdf]

nature portfolio

Corresponding author(s): Hong Han; Jason Moffat

Last updated by author(s): Aug 2, 2022

Reporting Summary

Nature Portfolio wishes to improve the reproducibility of the work that we publish. This form provides structure for consistency and transparency in reporting. For further information on Nature Portfolio policies, see our Editorial Policies and the Editorial Policy Checklist.

Statistics

For all statistical analyses, confirm that the following items are present in the figure legend, table legend, main text, or Methods section.

n/a	Confirmed	
☒	☐	The exact sample size (*n*) for each experimental group/condition, given as a discrete number and unit of measurement
☒	☐	A statement on whether measurements were taken from distinct samples or whether the same sample was measured repeatedly
☐	☒	The statistical test(s) used AND whether they are one- or two-sided<br>*Only common tests should be described solely by name; describe more complex techniques in the Methods section.*
☐	☒	A description of all covariates tested
☐	☒	A description of any assumptions or corrections, such as tests of normality and adjustment for multiple comparisons
☐	☒	A full description of the statistical parameters including central tendency (e.g. means) or other basic estimates (e.g. regression coefficient) AND variation (e.g. standard deviation) or associated estimates of uncertainty (e.g. confidence intervals)
☐	☒	For null hypothesis testing, the test statistic (e.g. *F*, *t*, *r*) with confidence intervals, effect sizes, degrees of freedom and *P* value noted<br>*Give P values as exact values whenever suitable.*
☒	☐	For Bayesian analysis, information on the choice of priors and Markov chain Monte Carlo settings
☒	☐	For hierarchical and complex designs, identification of the appropriate level for tests and full reporting of outcomes
☐	☒	Estimates of effect sizes (e.g. Cohen's *d*, Pearson's *r*), indicating how they were calculated

Our web collection on statistics for biologists contains articles on many of the points above.

Software and code

Policy information about availability of computer code

Data collection	No software was used for data collection. All publicly available datasets were obtained from the repository/source reported in the original publication (see data availability statement).
Data analysis	The computational tools developed in this study can be found at https://github.com/NMikolajewicz/scPipeline (scPipeline) and https://github.com/NMikolajewicz/scMiko (scMiko). Analyses performed in this study along with tutorial vignettes can also be found at https://nmikolajewicz.github.io/scMiko/. We also used the following open-source R packages to facilitate our analyses: Seurat (v 4.0.4) , Presto (v 1.0.0), chooseR (https://github.com/rbpatt2019/chooseR), fossil (v 0.4.0), igraph (v 1.2.6), NNLM (v 0.4.4), STRINGdb (v 2.0.2), fgsea (v 1.14.0), seqsetvis (v 1.8.0), ggVennDiagram (v 1.1.4), and ggplot2 (v 3.3.5).

For manuscripts utilizing custom algorithms or software that are central to the research but not yet described in published literature, software must be made available to editors and reviewers. We strongly encourage code deposition in a community repository (e.g. GitHub). See the Nature Portfolio guidelines for submitting code & software for further information.

Data

Policy information about availability of data

All manuscripts must include a data availability statement. This statement should provide the following information, where applicable:

- Accession codes, unique identifiers, or web links for publicly available datasets
- A description of any restrictions on data availability
- For clinical datasets or third party data, please ensure that the statement adheres to our policy

All data used for analysis and validation are available from the author's GitHub repository and public sources, Single cell RNA-seq data from Ochocka et al. (2021) was obtained from Gene Expression Omnibus (GEO; accession number GSE136001); Cao et al. (2019) from GEO (accession number GSE119945); Cao et al. 2020

March 2021

from GEO (accession number GSE156793); Zeisel et al. (2018) from http://mousebrain.org/downloads.html; La Manno et al. (2021) from http://mousebrain.org/downloads.html; Tabula Muris from FigShare; Tabula Sapiens from FigShare; Pijuan-Sala (2019) from the MouseGastrulationData R Package; and Tyser et al. (2021) from http://www.human-gastrula.net/.

Field-specific reporting

Please select the one below that is the best fit for your research. If you are not sure, read the appropriate sections before making your selection.

☒ Life sciences ☐ Behavioural & social sciences ☐ Ecological, evolutionary & environmental sciences

For a reference copy of the document with all sections, see nature.com/documents/nr-reporting-summary-flat.pdf

Life sciences study design

All studies must disclose on these points even when the disclosure is negative.

Sample size	No sample size calculations were performed for this study. To validate our computational tools, we identified 10 scRNAseq datasets comprised of diverse cellular populations (See Table 1 in manuscript for summary). The number of cell types in each scRNAseq data set ranged from 18-158, and the number cells in each data set ranged from 1,195-100,000.
Data exclusions	For larger public scRNAseq datasets (e.g., Cao 2020 comprised of 384615 cells), the number cells was randomly down-sampled to 100000 (See Table 1 in manuscript for summary of downsampled datasets). Otherwise, no data was excluded from analysis.
Replication	Our proposed methods were benchmarked using 8-10 scRNAseq datasets and we demonstrated consistent performance across each dataset, thereby supporting the reproducibility and generalizability of our methods. Importantly, performance was consistent between scRNAseq platforms (e.g., 10x, sci-RNAseq3, and SMART-seq2) and species (e.g., human and mouse).
Randomization	No randomization was performed in this study. To ensure covariates were controlled, we identified publicly available scRNA-seq atlases that were diverse and comprised of many distinct cell types, as opposed to using only homogenous scRNA-seq datasets in which biases may not be detected.
Blinding	No blinding was performed in this study. Blinding was not relevant to our study because we were not tested a specific hypothesis, but rather benchmarking our proposed computational methods.

Reporting for specific materials, systems and methods

We require information from authors about some types of materials, experimental systems and methods used in many studies. Here, indicate whether each material, system or method listed is relevant to your study. If you are not sure if a list item applies to your research, read the appropriate section before selecting a response.

Materials & experimental systems

n/a	Involved in the study	
☒	☐	Antibodies
☒	☐	Eukaryotic cell lines
☒	☐	Palaeontology and archaeology
☒	☐	Animals and other organisms
☒	☐	Human research participants
☒	☐	Clinical data
☒	☐	Dual use research of concern

Methods

n/a	Involved in the study	
☒	☐	ChIP-seq
☒	☐	Flow cytometry
☒	☐	MRI-based neuroimaging
